# The role of Volatile Anesthetics in Cardioprotection: a systematic review

**DOI:** 10.1186/2045-9912-2-22

**Published:** 2012-08-28

**Authors:** Nicole R Van Allen, Paul R Krafft, Arthur S Leitzke, Richard L Applegate, Jiping Tang, John H Zhang

**Affiliations:** 1 Department of Physiology, Loma Linda University School of Medicine, Risley Hall, Room 223, Loma Linda, CA 92354, USA; 2 Department of Anesthesiology, Loma Linda University School of Medicine, Loma Linda, CA, USA; 3 Department of Neurosurgery, Loma Linda University School of Medicine, Loma Linda, CA, USA

**Keywords:** Cardiac, Cardioprotection, Arrhythmias, Ischemia, Volatile anesthetic gas, Anesthesia

## Abstract

This review evaluates the mechanism of volatile anesthetics as cardioprotective agents in both clinical and laboratory research and furthermore assesses possible cardiac side effects upon usage. Cardiac as well as non-cardiac surgery may evoke perioperative adverse events including: ischemia, diverse arrhythmias and reperfusion injury. As volatile anesthetics have cardiovascular effects that can lead to hypotension, clinicians may choose to administer alternative anesthetics to patients with coronary artery disease, particularly if the patient has severe preoperative ischemia or cardiovascular instability. Increasing preclinical evidence demonstrated that administration of inhaled anesthetics - before and during surgery - reduces the degree of ischemia and reperfusion injury to the heart. Recently, this preclinical data has been implemented clinically, and beneficial effects have been found in some studies of patients undergoing coronary artery bypass graft surgery. Administration of volatile anesthetic gases was protective for patients undergoing cardiac surgery through manipulation of the potassium ATP (K_ATP_) channel, mitochondrial permeability transition pore (mPTP), reactive oxygen species (ROS) production, as well as through cytoprotective Akt and extracellular-signal kinases (ERK) pathways. However, as not all studies have demonstrated improved outcomes, the risks for undesirable hemodynamic effects must be weighed against the possible benefits of using volatile anesthetics as a means to provide cardiac protection in patients with coronary artery disease who are undergoing surgery.

## Introduction

Volatile anesthetics (VA) are gases that are used to induce and maintain general anesthesia, with benefits of relatively rapid onset and recovery with acceptable effects on peripheral organs in most patients. In 1275 Spanish physician Raymond Lullus made a volatile liquid that he called “sweet vitriol”—diethyl ether—which was later used as an anesthetic. Years later, an American physician, Crawford W. Long, noticed that colleagues under the influence of ether felt no pain when they injured themselves. In 1842, Long conducted the first surgery with the use of ether as an anesthetic, his patient, undergoing removal of a tumor in his neck, did not report pain. Long published his report in 1849, after William T. G. Morton had performed the first public demonstration of successful ether anesthesia in the operating amphitheater of Massachusetts General Hospital on October 16, 1846 [1].

VA have been shown to offer benefits in a wide range of medical situations such as use in stroke victims by reducing the amount of ischemic injury during the event of stroke and delaying the development of brain injury [2,3]; or as renal protection against ischemia-reperfusion injury—reducing plasma creatinine and reducing renal necrosis [4-9]. Some of these gases such as isoflurane, desflurane, and sevoflurane have demonstrated cardioprotection by reducing or preventing myocardial ischemia both intraoperatively and postoperatively [10]. However, VA administration is associated with myocardial depression and vasodilation that can contribute to intraoperative hypotension, potentially upsetting the balance between myocardial oxygen supply and demand with resulting intraoperative myocardial ischemia [5]. Thus many clinicians choose to limit or avoid administration of VA to patients undergoing coronary artery bypass surgery (CABG). For example nearly 40% of Italian heart surgery centers reported administration of VA to less than 25% of their CABG patients [11]. Similarly a meta-analysis found nearly half of patients were given total intravenous anesthesia in trials investigating VA effects during cardiac surgery [12]. Further, VA administration was found to be associated with worse outcome in the subset of cardiac surgery patients who had worse preoperative cardiac ischemia or cardiovascular instability [13]. Further, rapid induction of inhaled anesthesia can prolong the QT interval [14], which may be of concern in patients otherwise already at risk for ventricular fibrillation as may be seen during acute myocardial ischemia.

This review will focus on isoflurane, desflurane, and sevoflurane as agents used to achieve cardioprotection emphasizing recent laboratory and clinical data on the use of inhaled anesthetics for cardioprotection. It also discusses the possible cardiac risks associated with inhaled anesthesia.

### Literature search strategy

The literature search for this review focused on the volatile anesthetics isoflurane, desflurane, and sevoflurane. The following search conditions were used for each volatile anesthetic: volatile anesthetic AND cardiovascular protection, OR cardiac ischemia, OR cardiac injury, OR cardiac toxicity, OR cardiac ischemic preconditioning, OR myocyte toxicity, OR myocyte ischemia, OR myocyte hypoxia, OR cardiac hemorrhage, OR cardiac tolerance, OR cardiac postconditioning, OR cardiac preconditioning, OR myocyte apoptosis, OR cardiac arrhythmias. Articles that were not available in English were excluded from the references. The available literature is discussed as related to key areas of cardioprotection research and clinical care.

### Ischemic preconditioning

Ischemic injury is a pathological process that occurs when blood supply is interrupted to a specific area of tissue that occurs when oxygen (O_2_) demand exceeds O_2_ supply. In the event of ischemia, the myocardium continues to function by utilizing its stores of glycogen. However, if oxygen deprivation occurs for more than 15 minutes the myocardial tissue will become necrotic and lead to irreversible damage through tissue death [15]. Preconditioning is the process by which a certain level of injury is inflicted upon an organ or tissue, however this injury provides protection to the tissue in the event of a greater injurious process occurring. By inducing a short period of ischemia “cardiac myocytes reduce their contractile effort within a few seconds and stop contracting within the first few minutes” [16], which leads to energy conservation that helps protect myocardial tissue by reducing the amount of tissue necrosis. Cardiac ischemic preconditioning may lead to fatal consequences since impairment to the heart has immediate consequences to the rest of the body – leading to interruption of all organ system blood supplies which can result in brain damage, renal failure, pulmonary edema, etc. Clinical studies have shown that ischemic preconditioning reduces infarct size following induced myocardial ischemia [17-20]. This concept was first introduced in 1986 when Murry and colleagues demonstrated that ischemic preconditioning reduced infarct size in dogs from 29% in the control group to 7% in the group receiving ischemic preconditioning following coronary artery occlusion [20]. The dogs were subjected to brief periods of coronary artery occlusion (4-5 min) before an ischemic event consisting of a 40-min artery occlusion. The 22% reduction in infarct size observed by Murry et al. suggests that a protective mechanism underlies ischemic preconditioning.

**Figure 1:**
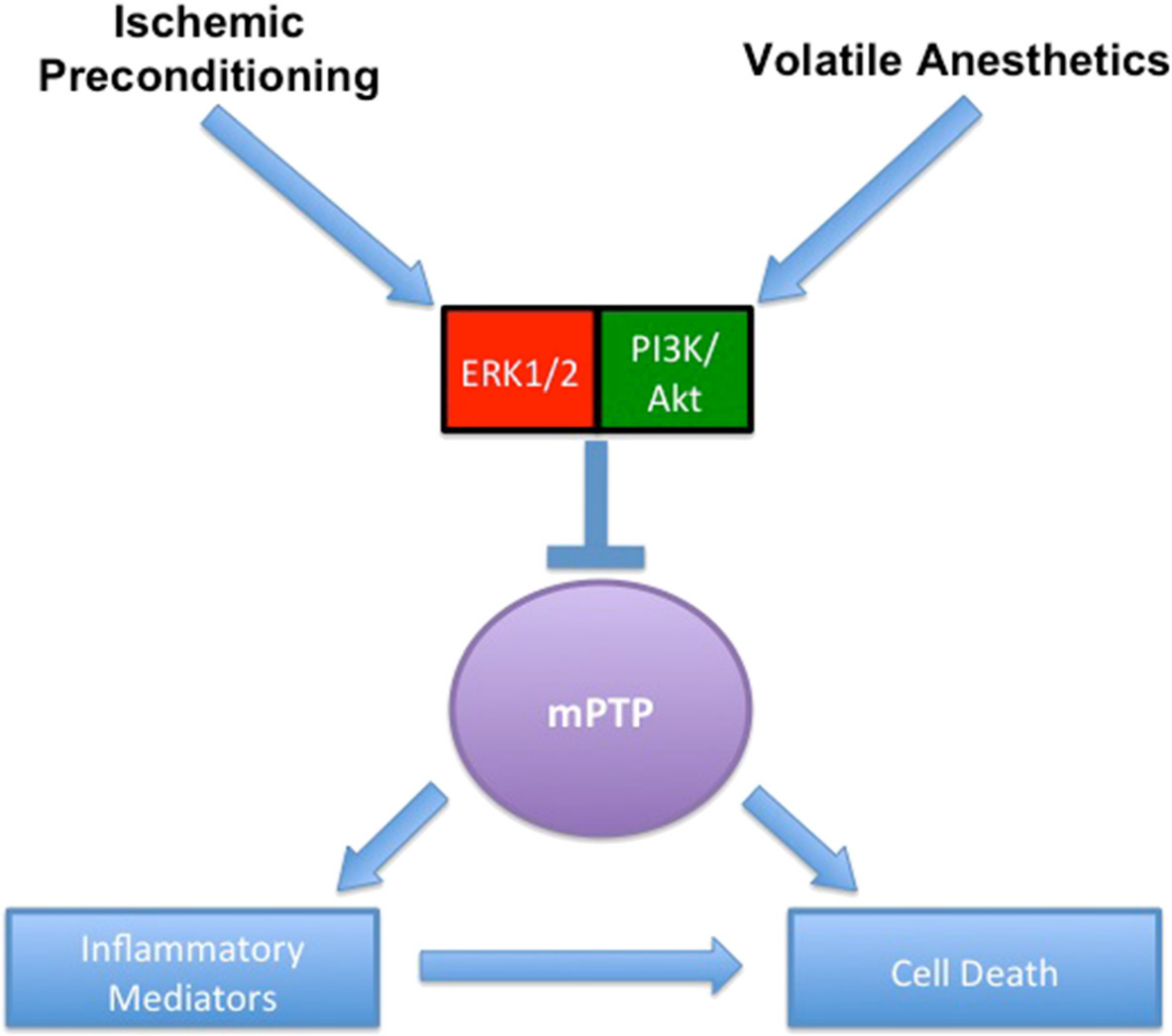
Mechanistic Effects of Ischemic Preconditioning and Volatile Anesthetic Pretreatment with Extracellular-signal Kinases (ERK), Cytokine Pathways (PI3K/Akt) and Mitochondrial Permeability Transition Pore (mPTP).

Additional research has linked several intracellular signaling pathways to the phenomenon of ischemic preconditioning. The primary target in all of these pathways appears to be the adenosine triphosphate (ATP)-sensitive K + (K_ATP_) channel. K_ATP_ channels are located on mitochondrial, sarcolemmal and nuclear membranes of cardiomyocytes, and are also found in the brain, pancreatic β cells, skeletal and smooth muscle, and neurons [21]. The opening of mitochondrial K_ATP_ channels leads to generation of reactive oxygen species (ROS), activating downstream kinases, resulting in cardioprotection [22] It has been shown that an initial increase in ROS leads to activation of the cytokine pathways such as protein kinase C (PKC) and tyrosine kinases (TK) which lead to opening of the mitochondrial K_ATP_ channels leading to a reduction in ROS. Thus an initial increase in ROS stimulated by ischemia leads to activation of pathways that result in reduction of ROS. In addition, activation and expression of K_ATP_ channels promotes action potential shortening and energy conservation, which is protective by preserving the cardiac tissue [23] Figure 1.

#### Reperfusion injury

Ischemic injury leads to reperfusion injury, when blood flow is restored to an ischemic area. The process of reperfusion leads to severe Ca^2+^ accumulation—due to cell membrane damage—that induces opening of the mitochondrial permeability transition pore (mPTP), which causes collapse of the mitochondrial membrane, uncoupling oxidative phosphorylation, resulting in ATP depletion and cell death. Ischemia also leads to conversion of xanthine dehydrogenase to xanthine oxidase in cardiomyocytes due to the lack of oxygen for metabolism. Xanthine oxidase leads to a build-up of hypoxanthine [24]. Upon reperfusion, xanthine oxidase metabolizes hypoxanthine, which leads to the overproduction of ROS. This phenomenon can cause already damaged tissue to produce superoxide radicals that further damage this tissue, leading to irreversible systolic dysfunction.

### Volatile Anesthetics pretreatment

The administration of anesthetics activates or primes some of the same pathways that lead to protection from ischemic preconditioning. Zaugg et al. [25] verified that exposing cardiomyocytes to volatile anesthetic (isoflurane or sevoflurane) prior to myocardial ischemia decreased ischemic damage in a dose-dependent manner similar to the beneficial effect from ischemic preconditioning, Through administration of K_ATP_ blockers 5-HD (mitochondrial K_ATP_ blocker) and HMR-1098 (sarcolemmal K_ATP_ blocker) as well as a K_ATP_ activator, diazoxide, they were able to show that isoflurane and sevoflurane prime the mitochondrial K_ATP_ channel but do not affect the sarcolemmal K_ATP_ channels. Zaugg and colleagues explained that sevoflurane and isoflurane administration lead to activation of mitochondrial K_ATP_ channels as is similarly seen in ischemic pretreatment. This same research group showed that outcome following coronary artery bypass surgery was improved by sevoflurane compared to placebo pretreatment, reducing the incidence of late cardiac ischemic and congestive heart failure [26]. However, it is not clear that the effect from volatile anesthesia pretreatment is additive to occlusion-induced ischemic preconditioning [27]. Warltier et al. demonstrated better recovery of myocardial function after 15 minutes of coronary artery occlusion when VA were administered prior to occlusion [28]. In this experiment dogs were anesthetized with halothane or isoflurane and their myocardial function returned to baseline within 5 hours after reperfusion. Dogs that did not undergo pretreatment with anesthesia experienced a 50% decrease in myocardial function. Further research has demonstrated a similar type of cardioprotection from ischemia and myocardial dysfunction using sevoflurane, desflurane and enflurane [29-31]. Piriou et al. [31] in a study on rabbit myocardium showed that desflurane is the most protective VA in pretreatment for ischemic injury whereas sevoflurane had no significant effect, and pretreatment with halothane and isoflurane induced the same cardioprotective effect. However, in other models sevoflurane pretreatment provides cardioprotection [32-39]

Additional mechanisms have been elicited that appear to be involved in cardioprotection as a result of preconditioning. These mechanisms include the Akt, ROS, and ERK pathways [32,34,39-49] (Table 1). Channels that have been identified to be involved are: mPTP, sarcolemmal K_ATP_ channel, and the mitochondrial K_ATP_ channel [25,33,45,48,50-85] (Table 2).

**Table 1:** Studies of the effects of Volatile Anesthetic Pretreatment relating to Akt, ROS, and ERK Mechanisms in cardioprotection

**Model**	**Anesthetic**	**Interventions**	**Mechanism**	**Length of ischemia**	**Infarct size**	**Ref #**
Isolated, perfused rat hearts with IR injury	Sevoflurane	Compound C, MPG	AMPK, eNOS	35 min	/	[24]
Isolated human right atrial trabeculae with IR injury	Sevoflurane & desflurane	MPG	ROS	30 min	/	[26]
Isolated rat ventricular cardiomyocytes with oxidative stress	Desflurane & sevoflurane	MPG	ROS	/	/	[31]
Global rabbit cardiac IR injury	Isoflurane	Wortmannin & LY204002	Akt & Bcl-2	40 min	41% control	[32]
					22% APT	
Isolated rat ventricular myocytes with hypoxia, hydrogen peroxide or neutrophil exposure	Isoflurane	H2O2 or neutrophils	Akt & Bcl-2	/	/	[33]
Inner mitochondrial membranes from isolated human left ventricles	Isoflurane	H2O2, ATP	ROS & KATP channel	/	/	[34]
Isolated right ventricular rat trabeculae with IR injury	Sevoflurane	KB-R7943 or SEA0400	PKC, Na+/Ca2+ channel	40 min	/	[35]
Isolated guinea pig hearts with IR injury	Sevoflurane	Superoxide dismutase, catalase & glutathione	ROS	30 min	49% control	[36]
					25% APT	
Isolated guinea pig ventricular myocytes	Isoflurane	N-acetyl cysteine, carnosine, superoxide dismutase, & catalase	ROS, sarcolemmal KATP channel	/	/	[37]
Isolated rat ventricular myocytes with IR injury	Isoflurane	MPG	Ca2+, ROS	30 min	/	[38]
Isolated guinea pig hearts with IR injury	Sevoflurane	Chelerythine, PP101, PP149	ROS, PKC	30 min	/	[39]
Isolated rat trabeculae with IR injury	Sevoflurane	Chelerythine, 5-HD, MPG	ROS, mPTP, PKC	30 min	/	[40]
Isolated rat trabeculae with IR injury	Sevoflurane	L-tyrosine, superoxide dismutase, glutathione, catalase, & L-NAME	ROS & NOS	30 min	50% control	[41]0
					18% APT	
Regional rat cardiac IR injury	Desflurane	Calphostin C, PD98059	PKC, ERK1/2	25 min	57% control	[42]
					35% APT	

Please note that “/” indicates that either this information was not printed or did not apply to the study. Ischemia-reperfusion (IR); Anesthetic Pretreatment (APT); Wortmannin & LY204002, PI3K inhibitors; hydrogen peroxide (H2O2); MPG, ROS scavenger; reactive oxygen species (ROS); Compound C, AMPK inhibitor; endothelial nitric oxide synthase (eNOS); KB-R7943 & SEA0400, Na+/Ca2+ channel inhibitors; N-acetyl cysteine, carnosine, superoxide dismutase & catalase (radical scavengers); PP101, PKC-δ inhibitor; chelerythrine (PP149), PKC-ϵ inhibitor; 5-HD, K_ATP_ channel blocker; mitochondrial permeability transition pore (mPTP); L-NAME, NOS inhibitor; calphostin C, PKC blocker; PD98059, ERK1/2 inhibitor.

**Table 2:** **Studies of the effects of Volatile Anesthetic Pretreatment relating to Mitochondrial Permeability Transition Pore and K**_**ATP **_**Channel Mechanisms in Cardioprotection**

**Model**	**Anesthetic**	**Interventions**	**Mechanism**	**Length of ischemia**	**Infarct size**	**Ref #**
Isolated rat ventricular myocytes	Sevoflurane & Isoflurane	5-HD, HMR-1098, diazoxide, chelerythine, 2,4-dinitrophenol	K_ATP_ activity	/	/	[17]
Isolated, perfused guinea pig hearts with IR injury	Sevoflurane	Chelerythine, 5-HD				[25]
Isolated guinea pig ventricular myocytes	Isoflurane	N-acetyl cysteine, carnosine, superoxide dismutase, & catalase	ROS, K_ATP_ activity	/	/	[37]
Isolated rate trabeculae with IR injury	Sevoflurane	Chelerythine, 5-HD, MPG	ROS, mPTP, PKC	30 min	/	[40]
Isolated, perfused rat heart with IR injury	Isoflurane	/	mPTP opening, O2 consumption	/	/	[43]
Isolated, perfused rat heart	Isoflurane	H2O2	mPTP opening, Ca2+	/	/	[44]
Regional rabbit cardiac IR injury	Desflurane	5-HD	mPTP, K_ATP_	10 min	/	[45]
Isolated mice hearts with IR injury	Isoflurane	/	mPTP opening	30 min	26% reduction from control	[46]
Global or regional rat cardiac IR injury	Isoflurane	/	mPTP opening	30 min	52% control	[47]
					30% APT	
Human right atrial appendages after anesthesia	Isoflurane	HMR-1098	K_ATP_ activity	15 min	/	[48]
Global or regional rat cardiac IR injury	Isoflurane	5-HD, TEMPO, L-NAME	K_ATP_ activity, NOS	30 min	62% control	[49]
					40% APT	
Isolated, perfused rat heart with cardioplegic arrest	Isoflurane	5-HD, HMR-1098	K_ATP_ activity	/	/	[50]
Isolated, perfused rat heart with IR injury	Isoflurane	5-HD, HMR-1098	K_ATP_ activity, Ca2+	30 min	/	[51]
Global rat cardiac IR injury	Isoflurane & sevoflurane	5-HD	K_ATP_ activity	10 min	/	[52]
Isolated rat cardiomyocytes with oxidative stress	Isoflurane	H202, FeSO4, 5-HD, HMR-1098	K_ATP_ activity	/	/	[53]
Rat ventricular cardiomyocytes with patch-clamp	Isoflurane	/	K_ATP_ activity	/	/	[54]
Isolated, rat ventricular trabeculae	Sevoflurane	Chelerythrine, 5-HD, MPG	K_ATP_ activity, PKC, ROS	30 min	/	[55]
Global rat cardiac IR injury	Sevoflurane	5-HD	K_ATP_ activity	25 min	/	[56]
Isolated, rat ventricular cardiomyocytes	Isoflurane	Chelerythrine, nisoldipine, glibenclamide	K_ATP_ activity & PKC	/	/	[57]
Isolated guinea pig hearts	Sevoflurane	5-HD	K_ATP_ activity	2 hours	/	[58]
Isolated guinea pig hearts with patch-clamp	Isoflurane	PP106 (PKC activator), PP93 (PKC antagonist), PP1144 (PKC act), 5-HD	K_ATP_ activity	/	/	[59]
Isolated, perfused rat hearts with IR injury	Isoflurane	5-HD, diazoxide	K_ATP_ activity, O2-	30 min	37% control	[60]
					24% APT	
Isolated, guinea pig ventricular myocytes with patch-clamp	Isoflurane	ATP, pinacidil, 2,4,-dinitrophenol, glibenclamide	K_ATP_ activity	/	/	[61]
Isolated, perfused guinea pig hearts with IR injury	Sevoflurane	MnTBAP, 5-HD	K_ATP_ activity, ROS	30 min, 5–20 min	/	[62]
Global or regional Rat cardiac IR injury	Isoflurane	Glibenclamide	K_ATP_ activity, PKC	/	58% control	[63]
					42% APT	
Isolated guinea pig ventricular cardiomyocytes	Isoflurane & halothane	Pinacidil, 2,4-dinitrophenol	K_ATP_ activity	/	/	[64]
Isolated, perfused guinea pig ventricular myocytes	Isoflurane	ADP, nisoldipine, 2,4-dinitrophenol, ATP, adenosine, GTP	K_ATP_ activity, PKC	/	/	[65]
Isolated guinea pig ventricular myocytes	Isoflurane	Genistein & tryphostin B42	Tyrosine kinase depend K_ATP_	/	/	[66]
Isolated, perfused rat hearts with IR injury	Isoflurane	Glyburide	K_ATP_ activity	15 min	/	[67]
Isolated human right atrial trabeculae with IR injury	Desflurane	Glibenclamide, phentolamine, propranolol, DCPCX, 5-HD & HMR-1098	K_ATP_ activity, adrenergic role	30 min	/	[68]
Isolated guinea pig hearts with IR injury	Sevoflurane	5-HD	K_ATP_ activity	4 hours	36% control	[69]
					25% APT	
Isolated, perfused rabbit hearts with IR injury	Isoflurane	5-HD, HMR-1098	K_ATP_ activity	/	20% control	[70]
					10% APT	
Participle, isolated rat hearts with IR injury	Isoflurane	5-HD	K_ATP_ activity	20 min	/	[71]
Global Dog cardiac IR injury	Sevoflurane	ATP, 5-HD	K_ATP_ activity	15 min	/	[72]
Isolated, perfused human atrial trabeculae	Isoflurane & halothane	Glibenclamide, DPCPX	K_ATP_ activity	60 min	/	[73]
Isolated, perfused rat hearts with IR injury	Sevoflurane & halothane	Glibenclamide	K_ATP_ activity,	45-60 min	/	[74]
Isolated, perfused rat hearts	Sevoflurane	Pinacidil, glyburide	K_ATP_ activity	60 min	/	[75]
Regional Dog cardiac IR injury	Isoflurane	Glyburide	K_ATP_ activity	5 min, x 5	/	[76]
Regional Dog cardiac IR injury	Isoflurane	Glibenclamide	K_ATP_ activity	15 min	/	[76]

Please note that “/” indicates that either this information was not printed or did not apply to the study. 5-HD, K_ATP_ inhibitor; HMR-1098, sarcolemmal K_ATP_ inhibitor; diazoxide & pinacidil, K_ATP_ activators; chelerythrine, PKC-ϵ inhibitor; N-acetyl cysteine, carnosine, superoxide dismutase & catalase (radical scavengers); reactive oxygen species (ROS); potassium ATP channel (K_ATP_ ); Anesthetic Pretreatment (APT); ischemia-reperfusion (IR); mitochondrial permeability transition pore (mPTP); hydrogen peroxide (H2O2); inducible nitric oxide synthase (iNOS); TEMPO, O2 scavenger; L-NAME, NOS inhibitor; ferrous sulfate (FeSO4); MPG, ROS scavenger; nisoldipine, L-type Ca2+ channel blocker; glibenclamide, K_ATP_ inhibitor; PP106 & PP1144, PKC activators; PP93, PKC inhibitor; adenosine triphosphate (ATP); 2,4-dinitrophenol, ATP production inhibitor; MnTBAP, radical scavenger; adenosine diphosphate (ADP); guanosine triphosphate (GTP); genestein & tryphostin B42, tyrosine kinase inhibitors; glyburide, K_ATP_ inhibitor; phentolamine, α blocker; propranolol, β blocker; DCPCX, adenosine A1 antagonist.

#### Mechanistic pathways

Several key mechanistic pathways and regulators that have been identified as mediators in the protective effects of pretreatment with VAGs including K_ATP_ channel activation, mPTP modulation and the cytokine pathways (Akt/PI3K). K_ATP_ channels, established as cardioprotective mediators in ischemic preconditioning, have been studied in VA pretreatment. It has been shown that opening of the mitochondrial K_ATP_ channel leads to generation of ROS [22]. In a study utilizing rat trabeculae, de Ruijter et al. [48] demonstrated that the cardioprotective effect of sevoflurane occurs via the activation of PKC, which leads to mitochondrial K_ATP_ channel opening. Rat trabeculae underwent ischemia and then 60 minutes of reperfusion, and the recovery of active force was used as a measure of cardiac function following myocardial infarction (MI). Sevoflurane improved recovery of active force to 67% as opposed to 28% in the control group. However, when the K_ATP_ channel inhibitor (5-HD) was administered along with sevoflurane force recovery was only 31%, while the administration of a ROS scavenger and sevoflurane resulted in force recovery of only 33%. This data indicates that both the K_ATP_ channel and ROS are involved in the protective mechanisms of sevoflurane. According to Marinovic et al. [60] it appears that the sarcolemmal K_ATP_ channel is an effector of pretreatment whereas mitochondrial K_ATP_ channels are both a trigger and an effector. This was established when both mitochondrial and sarcolemmal K_ATP_ channel inhibitors were administered during isoflurane pretreatment in rat cardiomyocytes and a reduction in protection from the sevoflurane pretreated group was observed with 5-HD but not with HMR-1098. However, if HMR-1098 was applied throughout the experiment rather than during just pretreatment, the protective effect was abolished.

Piriou et al. [52] noted that the K_ATP_ channel is also linked to the mPTP. With this study it was suggested that both ischemic preconditioning and VA pretreatment delays mPTP opening. Delaying mPTP opening is protective because opening of the mPTP leads to swelling of the mitochondrial matrix, which causes collapse of the inner mitochondrial membrane, uncoupling of the electron transport chain, and release of cytochrome c along with other apoptotic factors such as Bax, caspase-9 and ATP. Administering 5-HD abolished the improved tolerance to calcium-induced mPTP opening. This demonstrates a likely connection between the mPTP and K_ATP_ channel.

Another pathway that has been identified clinically in cardioprotection is the Akt/PI3k pathway, which is a key intracellular signaling pathway in apoptosis. Raphael et al. [40] studied the role of the Akt/PI3K pathway in VA cardioprotection. Examination of DNA fragmentation conducted using the TUNEL method demonstrated that isoflurane pretreatment significantly reduced the percentage of apoptotic nuclei. Furthermore, evaluation of the Akt and phosphorylated-Akt (active Akt) expression during ischemia and reperfusion revealed that phosphorylated Akt was expressed in significantly higher numbers for the ischemia-reperfusion and isoflurane pretreated groups while administration of wortmannin and LY294002 (PI3K inhibitors) led to inhibition of phosphorylated Akt. Further, treatment with wortmannin and LY294002 abolished the cardioprotective effects of anesthetic pretreatment, indicating that phosphorylated Akt leads to cardioprotection.

The extracellular-signal kinases (ERK) pathway has been linked to myocardial protection elicited by pretreatment with VA. Toma et al. [86] studied ERK phosphorylation (activated form of ERK) that was induced by pretreatment with desflurane; rats were subjected to myocardial ischemia and reperfusion. Administration of the MEK/ERK1/2 inhibitor PD98059 with desflurane eliminated cardioprotection that was observed in the desflurane-only pretreatment group. This points to MEK/ERK1/2 as modulators of the protective effects of VA administration prior to injury. Western blot analysis showed an early increase in ERK phosphorylation with the first administration of desflurane 10 minutes post-MI. However, this decreased with the second desflurane dose 25 minutes post-MI. Even though it has been shown that ERK1/2 is a downstream effector of PKC mediating effects it was discovered that ERK phosphorylation was not PKC dependent. Giving rats a dose of calphostin C (PKC inhibitor) did not affect the phosphorylation of ERK1/2 observed on Western blot. These results illustrate ERK1/2 activation as cardioprotective with a single dose of desflurane but this cardioprotection is diminished with an additional dose, highlighting the importance of administration protocols for VA pretreatment. Finally, it was indicated that ERK1/2 activation is independent on PKC.

In addition, Ca^2+^ flux has been linked to cardioprotection via VA pretreatment as well as nuclear factor-κB (NF-κB) involvement. An et al. [87] measured Ca^2+^ concentration by fluorescence and demonstrated that pretreatment with sevoflurane improved coronary blood flow and reduced systolic Ca^2+^ loading. Further, decreased destruction of sarcoplasmic reticulum Ca2+ − cycling proteins was observed on Western Blot. The reduced systolic Ca^2+^ leads to the conclusion that this is a cardioprotective effect since reperfusion injury—which leads to irreversible damage—is a result of Ca^2+^ excess. The accumulation of Ca^2+^ after ischemia-reperfusion leads to activation of NF-κB, which causes release of inflammatory mediators. Further research demonstrated preservation of calcium cycling proteins following VA pretreatment in a myocardial ischemia-reperfusion model [58]. Konia et al. [88] studied the inhibition of NF-κB in rats pretreated with sevoflurane; parthenolide (IF-κB inhibitor) was administered to prevent activation of NF-κB. It was concluded that inhibition of NF-κB leads to even greater protection from ischemia than sevoflurane administration alone; sevoflurane treatment group exhibited an infarct size of 19%, parthenolide group exhibited an infarct size of 18%, the sevoflurane + parthenolide group exhibited an infarct size of 10%, compared to the control groups with an infarct size of 59%. The involvement of NF-κB for anesthetic pretreatment needs to be further examined and directly linked to anesthesia pretreatment.

#### Clinical research

While anesthetic pretreatment demonstrates cardioprotection in the laboratory, it is crucial to answer the question whether these cardioprotective effects are also clinically applicable. Cardiac surgery is a suitable model for studying VA pretreatment, however the administration of other anesthetics during the cardiac surgery may also provide protection, leading to difficulty of clearly answering the question of whether VA are cardioprotective clinically. Clinical trials have evaluated VA pretreatment on patients undergoing cardiac surgery - especially CABG, some of which support a beneficial effect of VA for decreasing myocardial infarction, troponin release, hospital length of stay and death [12,89,90].For example, in studies involving CABG patients, Guarracino et al. [91] and Meco et al. [92] (Meco 2007) found that desflurane administration was associated with lesser postoperative elevation of biochemical markers of myocardial injury than total intravenous anesthesia. In contrast, De Hert et al. did not find a difference in postoperative biochemical markers of myocardial injury in patients given desflurane or sevoflurane compared to those receiving total intravenous anesthesia. However, patients given either VA had shorter hospital length of stay and lower 1-year mortality [93]. In a retrospective study including over 10,000 cardiac surgery patients, VA administration was associated with better outcomes in patients undergoing elective cardiac surgery. However, in patients with severe preoperative myocardial ischemia or cardiovascular instability, the administration of VA was associated with worse outcome than the administration of total intravenous anesthesia [13]. Added evidence to support a benefit to the use of VA in cardiac surgery was reported by Bignami et al. [11] who found better outcomes following cardiac surgery in centers in which cardiac surgery patients are given VA. This analysis suggested the benefit was greater when VA are given for a greater portion of the procedure. Amr et al. found both ischemic preconditioning and isoflurane preconditioning were associated with better cardioprotection than cold blood cardioplegia in CABG patients anesthetized with total intravenous anesthesia [94]. Further evidence to support a beneficial effect of VA administration to CABG patients is that remote ischemic preconditioning was associated with benefit in patients anesthetized with VA but not in those given propofol for anesthesia [95]. An international consensus conference provided expert opinion support for the use of VA in hemodynamically stable cardiac surgery patients [96] as a means to reduce myocardial damage and death. This consensus concluded that the further large randomized controlled trials of VA administration to cardiac surgery patients are necessary.

Several studies have used human cardiac tissue to examine the benefits of VA pretreatment as well as to identify similar mechanistic pathways involved to those elicited in animal studies. Some key mechanistic pathways have been examined using drugs that antagonize ion channels and pathways involved in anesthesia pretreatment. Jiang et al. [42] used human ventricular muscle cell not suitable for donor transplantation, to examine the presence of mitochondrial K_ATP_ channel activity in human tissue. By providing a dose of 5-HD to cells it was demonstrated that modulation of the mitochondrial K_ATP_ channel is involved in human as well as animal cardiomyocytes during ischemic injury. Administration of 5-HD reduced mitochondrial K_ATP_ channel activity in the treatment group. In another group, isoflurane increased mitochondrial K_ATP_ channel activity and increased peak current beyond that of the control group demonstrating the role of K_ATP_ channel in VA pretreatment clinically. Further clinical studies have shown that ROS are involved in cardioprotection from anesthetic pretreatment; therefore, they investigated the effects of exogenous hydrogen peroxide (H_2_O_2_) in their apparatus. First, a cluster of mitochondrial K_ATP_ channels were suppressed by ATP administration, next H_2_O_2_ was given which resulted in a reactivation of the K_ATP_ channels despite the continued presence of ATP indicating that ROS influences K_ATP_ channels in human myocardium, *in vitro*.

In an *in vitro* study using right atrial appendages obtained from adult patients undergoing cardiac surgery, Mio et al. [55] explored the mechanistic effects of VA pretreatment. They suggested that K_ATP_ channels are involved in cardioprotection from pretreatment with volatile anesthetics, as their results demonstrated isoflurane decreased stress-induced cell death and maintained mitochondrial function. Isoflurane preserved mitochondrial oxygen consumption which was initiated by pyruvate-malate and accelerated by adenosine diphosphate (ADP). Preservation of mitochondrial oxygen consumption indicates a cardioprotective effect from isoflurane. In addition, they noted that isoflurane was protective via the sarcolemmal K_ATP_ mechanism. Administration of HMR-1098 diminished the cardioprotective effect of isoflurane from a cell death percentage of 21% (without HMR-1098) to a cell death percentage of 41% with HMR-1098 indicating the involvement of K_ATP_ channel clinically.

Hanouz et al. [34] studied the role of ROS in cardioprotection from pretreatment with sevoflurane and desflurane by using *in vitro* human right atrial trabeculae. Recovery of force of contraction was studied in each experimental group: control, sevoflurane pretreatment, and desflurane pretreatment. Force of contraction recovery was significantly improved in the sevoflurane group (from 53% to 85%) and in the desflurane group (from 53% to 86%). Treatment with MPG (ROS scavenger) prevented the force of contraction recovery: in the desflurane + MPG group, force of contraction changed from 53% to 48% and in the sevoflurane + MPG group force of contraction changed from 53% to 56% (both the same as control). Since the administration of MPG abolished recovery in both the desflurane and sevoflurane groups, they concluded that ROS must play a role in the cardioprotective mechanisms triggered by VA pretreatment. The mechanistic pathways of cardioprotection in human tissue has been evaluated through *in vitro* studies, however further *in vivo* studies are necessary to definitively establish VA pretreatment as a treatment option for cardioprotection in patients at-risk for myocardial ischemia.

### Cardiac side effects of medical gas anesthesia

All VA have clinically relevant myocardial depressant effects when given in usual anesthetic concentrations [97]. These effects may contribute to the cardioprotective effects of VA, but must be considered when administering VA to patients with significant cardiac dysfunction. In addition to myocardial depressant effects, VA administered in clinically relevant concentrations cause vasodilation, which can contribute to hemodynamic instability when given to patients with ischemic cardiac disease. Additionally, several studies have demonstrated that administration of VA may lead to prolongation of the QT interval [98-105]. This is concerning because a prolonged QT interval increases the risk for arrhythmia development. The QT interval is the portion on an electrocardiogram representing the portion of the cardiac electrical cycle in which both depolarization and repolarization of the ventricles occur. Prolonging the length of this interval increases a patient’s risk for *torsades de pointes,* which may lead to ventricular fibrillation [106,107] as has been reported during anesthetics with VA [108-110]. Despite this, VA use has been reported as safe in patients with known long QT syndrome [111,112]. Furthermore, even though studies have demonstrated that VA administration prolongs the QT interval, ventricular arrhythmia incidence was lower in the subset of 10,535 CABG patients who were given sevoflurane compared to those given propofol for anesthesia [13]. Other studies of patients undergoing CABG did not report an increase in ventricular arrhythmias following pretreatment with VA [4,26,113]. Further, animal studies show that VA pre- or post-conditioning provides an antiarrhythmic effect [114-116]. However, in patients whose medical condition may predispose to cardiac dysrhythmia or hemodynamic instability, such as those with severe preoperative myocardial ischemia, it is reasonable to use greater caution when administering VA for cardiac protection (Table 3).

**Table 3:** Studies of the effects of Volatile Anesthetic pretreatment relating to QT changes and arrhythmias

**Model**	**Drug(s)**	**Techniques**	**Mechanism**	**Ref #**
General anesthesia	Sevoflurane & propofol	ECG	Pwt, QTc intervals & QT	[85]
General anesthesia	Sevoflurane	ECG	Qt interval	[86]
General anesthesia	Sevoflurane	ECG	QT interval, Tp-e interval	[87]
General anesthesia	Desflurane, sevoflurane, propofol	ECG	QT dispersion	[88]
General anesthesia	Sevoflurane & desflurane	ECG	QTc intervals	[89]
General anesthesia	Sevoflurane	ECG	QT intervals	[90]
General anesthesia	Sevoflurane	ECG	QT, QTc, TDR, TdP, Tp-e intervals	[91]
General anesthesia	Sevoflurane	ECG	QT interval	[92]

#### Lack of cardioprotection?

Zangrillo et al. [117] recently demonstrated that no cardioprotection exists with pretreatment of VA in non-cardiac surgeries. This study suggests that non-cardiac surgery patients do not receive any reduction in release of troponin postoperatively (myocardial injury marker). Similarly, Piriou et al. [118] did not find significant effects of VA in a randomized trial of patients undergoing CABG, while De Hert et al. [93] in a multicenter randomized trial studying over 400 patients found no differences in markers of cardiac damage following CABG in patients given VA compared to intravenous anesthesia. Bignami et al. did not find benefit from sevoflurane administration to patients with known coronary artery disease who were undergoing mitral valve surgery [119]. A meta-analysis of studies enrolling over 6,200 patients undergoing noncardiac surgery did not find myocardial infarction or death in the studies reviewed, which limited analysis of choice of anesthetic technique impact on clinically relevant outcomes [120]. However, Bassuoni et al. reported a beneficial effect of VA administration compared to propofol anesthesia [121]. They found VA administration was associated with less myocardial ischemia and troponin release after noncardiac peripheral vascular surgery in a study of 126 patients who did not have significant additional comorbid conditions.Recommendations from the American College of Cardiology/American Heart Association guidelines that state that patients at risk for myocardial infarction during non-cardiac surgery would benefit from pretreatment with gas anesthesia, if hemodynamically stable [122].

### Conclusion and future direction

Abounding evidence has shown that pretreatment with volatile anesthetics protects against ischemia/reperfusion injury in both animal and clinical studies. Future investigations need to evaluate the most optimal anesthetic agent, concentration and administration protocol for the best cardioprotective benefits of pretreatment, as studies have noted that there is a difference in cardioprotection dependent upon pretreatment protocol with VA [11]. Additionally, a comprehensive mechanistic model needs to be elicited that integrates all mechanisms evaluated thus far. Several other players in the mechanism of cardioprotection have been examined. However, further studies are needed to confirm these mechanisms, which include: caveolin [123], caspase [124], Pim-1 kinase [125], β-adrenergic [126], and coronary vasodilation [127,128].

We conclude the results of laboratory studies provide mechanistic pathways supporting the cardioprotective effect of pretreatment with VA. Our opinion is these effects should be beneficial to patients with ischemic cardiac disease who are undergoing surgery. However, the optimum dose and timing of VA administration for this effect must be further investigated. It is reasonable to use greater caution when administering VA for cardiac protection to patients in whom preoperative hemodynamic conditions or cardiac rhythm would magnify the known side effects of VA (vasodilation, cardiac depression or QT prolongation).

## Abbreviations

ATP: Adenosine triphosphate -sensitive K + (K_ATP_) channel; mPTP: Mitochondrial permeability transition pore; ROS: Reactive oxygen species; VA: Volatile anesthetics; O_2_: Oxygen; ATP: Adenosine triphosphate; PKC: Protein kinase C; TK: Tyrosine kinase; FoC: Recovery of force of contraction; MI: Myocardial infarction; ERK: Extracellular-signal kinases (ERK) pathway; NF-κB: Nuclear factor-κB; CABG: Coronary Artery Bypass Graft; H_2_O_2_: Hydrogen peroxide; ADP: Adenosine diphosphate; ECG: Electrocardiogram.

## Competing interests

The authors declare they have no competing interests.

## Authors’ contribution

NV-Role included reviewing manuscripts, review design, and manuscript preparation. PK-Role included review design and manuscript editing. AL-Role included literature search, review design and proof reading. RA-Role included editing and manuscript preparation. JT-Role included manuscript proof reading. JZ-Role included review design and manuscript proof reading. All authors read and approved the final manuscript.
